# Thermophysical Investigation of Multiform NiO Nanowalls@carbon Foam/1-Octadecanol Composite Phase Change Materials for Thermal Management

**DOI:** 10.3390/molecules29184453

**Published:** 2024-09-19

**Authors:** Xiuli Wang, Qingmeng Wang, Xiaomin Cheng, Wen Xiong, Xiaolan Chen, Qianju Cheng

**Affiliations:** 1 School of Mechatronics and Intelligent Manufacturing, Huanggang Normal University, Huanggang 438000, China; wangxiuli@whut.edu.cn (X.W.); chengxm@whut.edu.cn (X.C.);; 2 School of Materials Science and Engineering, Wuhan University of Technology, Wuhan 430070, China

**Keywords:** multiform NiO, carbon foam, PCMs, thermal management

## Abstract

Multiform NiO nanowalls with a high specific surface area were constructed in situ on carbon foam (CF) to construct NiO@CF/OD composite phase change materials (CPCMs). The synthesis mechanism, microstructures, thermal management capability, and photothermal conversion of NiO@CF/OD CPCMs were systematically studied. Additionally, the collaborative enhancement effects of CF and multiform NiO nanowalls on the thermal properties of OD PCMs were also investigated. NiO@CF not only maintains the porous 3D network structure of CF, but also effectively prevents the aggregation of NiO nanosheets. The chemical structures of NiO@CF/OD CPCMs were analyzed using XRD and FTIR spectroscopy. When combined with CF and NiO nanosheets, OD has high compatibility with NiO@CF. The thermal conductivity of NiO@CF/OD-L CPCMs was 1.12 W/m·K, which is 366.7% higher than that of OD. The improvement in thermal conductivity of CPCMs was theoretically analyzed according to the Debye model. NiO@CF/OD-L CPCMs have a photothermal conversion efficiency up to 77.6%. This article provided a theoretical basis for the optimal design and performance prediction of thermal storage materials and systems.

## 1. Introduction

Thermal management materials are used to enhance heat conduction efficiency and evenly disperse heat, and are indispensable materials in fields such as consumer electronics, automotive electronics, and communication equipment [1,2,3]. Phase change materials (PCMs) can realize heat dispersion, storage, and conversion control with their high heat storage capacity, and have broad application prospects in heat storage, chip heat dissipation, heat storage, and insulation [4,5]. Organic PCMs have stable thermal properties, but their low thermal conductivity limits their large-scale application [6,7]. In response to the requirements of precise temperature control applications, the question of how to significantly improve the energy storage density and charging/discharging efficiency of thermal storage materials is the current research focus.

Carbon materials can be used as high thermal conductivity modifiers and porous support materials for PCMs because of their porous structure, high thermal conductivity, good chemical stability, strong plasticity, easy access, low density, and large specific surface area [8,9,10]. Singh et al. [11] compared the preparation and properties of carbon-based organic PCMs, and the carbon materials were effective in enhancing the thermal properties of PCMs. Zhao et al. [12] prepared GA/PEG CPCMs by the adsorption of PEG with GA, in which the mass fraction of PEG was as high as 96.0%, the latent heat of melting was 223.2 J/g, the latent heat of solidification was 218.8 J/g, and the highest thermal conductivity was 0.36 W/(m·K). To reduce the cost of using carbon-based materials in the field of phase change energy storage and to improve their economy, a new approach has been provided for utilizing low-cost and renewable biomass carbon materials. Nagar et al. [13] introduces an innovative paraffin/carbon foam CPCM heat sink for efficient thermal management of building-integrated photovoltaic panels. The proposed heat sink reduced the panel temperature to 23 °C. YAN et al. [14] prepared OC/EG/CuS@ZnO by skillfully combining highly adsorbable MOFs with EG and reacting octadecanol (OC). The latent heat of melting of the OC/EG/CuS@ZnO was 202.97 J/g and the thermal conductivity was 11.77 W/(m·K) when m(ZnO):m(EG) = 1:8. It was verified that OC/EG/CuS@ZnO possesses strong heat dissipation capability and can be applied in solar thermal storage and electronic component heat dissipation. Aljafari et al. [15] systematically analyzed the enhanced thermal characteristics of graphene (Gr) and Ag nanoparticles on the energy storage performance of PCMs. The results showed that the thermal conductivity of PCMs increased by 96% when 0.8% Gr:Ag was added. The above research indicates that composite thermal storage materials with high thermal storage density, high thermal conductivity, and specific heat capacity can be obtained through organic–inorganic composites [16,17]. Currently, most research focuses on the composite preparation and performance characterization of thermal storage materials [18,19]. Although the obtained thermal storage materials have a certain degree of improvement in thermal storage performance, there is still no controllable preparation principle and composite method that can significantly enhance thermal storage density and thermal conductivity [20]. Moreover, there is limited exploratory research on the heat storage and release mechanisms of organic–inorganic CPCMs.

NiO belongs to the transition group of metal oxides, with specific photoelectric properties and important applications in photocatalysis, magnetism, and optics, etc. [21,22]. Huang et al. [23] used a simple thermal decomposition method to generate uniformly and vertically distributed nano NiO from a three-dimensional foam nickel matrix. The high-conductivity foam nickel was used as the supercapacitor electrode material, which has good cycling stability. There have been few studies on using nano NiO to enhance the thermal properties of organic phase change thermal storage materials. Based on the composite design idea of the structure and function of PCMs, in this study, we combine the size effect of NiO nanowalls and the high thermal conductivity of CF to construct a multicomponent high thermal conductivity framework for improving the heat storage and exchange performance of 1-octadecanol (OD) PCMs through component modulation and process optimization. The composition, microstructure, thermal properties, and performance stability of the CPCMs were studied systematically. The relationship between the microstructure and thermal storage properties of CPCMs was established. In addition, the mechanism of influence of NiO@CF on the thermal properties of CPCMs was discussed.

## 2. Results and Discussion

### 2.1. Microstructure

**Figure 1:**
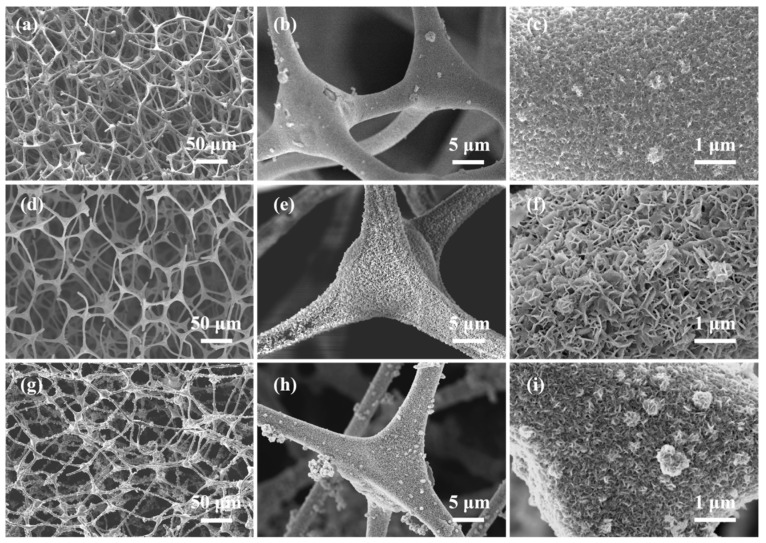
SEM images of (**a**–**c**) NiO@CF-P, (**d**–**f**) NiO@CF-L, and (**g**–**i**) NiO@CF-S.

Figure 1 shows the microscopic morphology of NiO@CF. The NiO nanolayer grows uniformly on the surface of the fiber after 350 °C calcination and maintains a 3D network structure, which indirectly proves the strong mechanical strength of CF. From Figure 1a–c, it can be seen that NiO is densely covered on the NiO@CF-P skeleton in the form of nanoparticles, and a small number of NiO nanoparticle layers were exfoliated. For NiO@CF-L, NiO is uniformly distributed as nanowalls on the surface of CF fibers, NiO nanosheets are uniform in size without obvious agglomeration, and the size of a single nanosheet is about 300 nm with no secondary growth. NiO nanosheets on the NiO@CF-S skeleton undergo severe secondary growth, and the secondary growth of NiO exists as nanospheres. The generation of NiO nanosheets substantially increases the overall specific surface area of the thermally conductive enhanced phase. Each lamella still has a microporous structure with a nanoscale size, which further increases the specific surface area of the sample, and the mutual structure of the two forms a novel 2D/3D hybrid structure.

**Figure 2:**
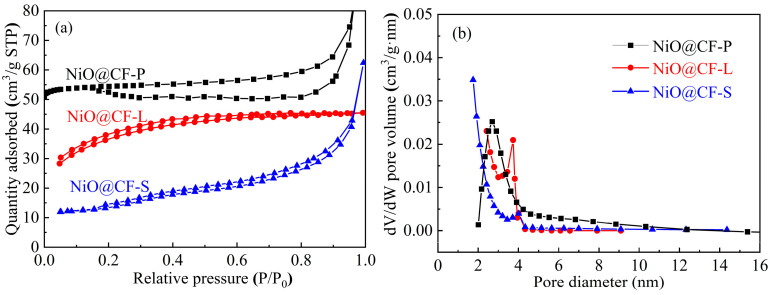
(**a**) N_2_ adsorption–desorption isotherms and (**b**) pore size distribution of NiO@CF.

The specific surface area and pore size distribution of NiO@CF with different morphologies were tested, and the results are shown in Figure 2. Compared with the classification of nitrogen adsorption and desorption isotherms by the international organization IUPAC, the adsorption and desorption isotherms of NiO@CF-P, NiO@CF-L, and NiO@CF-S belong to type IV isotherms [24,25]. From Figure 2a, hysteresis loops can be observed, which means that the isotherms obtained during the desorption process of the three samples do not completely overlap with the isotherms of the adsorption process. In the area where the relative pressure increases, the desorption isotherms are located above the adsorption isotherms, and the appearance of hysteresis loops is related to the capillary condensation process. The specific surface area, average pore diameter, and pore volume of the samples are listed in Table 1. The specific surface areas of NiO@CF-P, NiO@CF-L, and NiO@CF-S were 176.2 m^2^/g, 101.9 m^2^/g, and 80.5 m^2^/g, and the average pore sizes were 5.69 nm, 2.86 nm, and 2.41 nm, and the pore volumes were 0.09 cm^3^/g, 0.07 cm^3^/g, and 0.05 cm^3^/g, respectively. Combined with Figure 2b, it can be seen that NiO@CF-P has a larger specific surface area and a more concentrated pore size distribution, and the average pore size and pore volume are higher than those of NiO@CF-L and NiO@CF-S. The large specific surface area and uniform pore size of NiO@CF can stabilize the storage of liquid OD and prevent leakage, contributing to the thermal stability of the composite phase change thermal storage material.

### 2.2. Component

**Figure 3:**
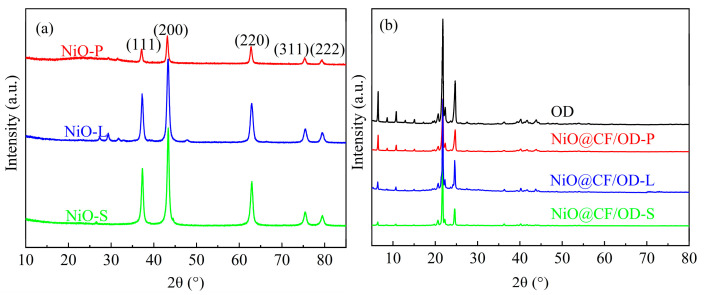
XRD spectra of (**a**) NiO and (**b**) OD and NiO@CF/OD CPCMs.

Figure 3 shows the XRD plots of OD, NiO, and NiO@CF/OD CPCMs. The diffraction peaks of NiO nanoparticles isolated from the three samples are located at 37.1°, 43.2°, 63.0°, 75.5°, and 79.5°, which correspond to the (111), (200), (220), (311), and (222) planes of the cubic phase NiO (JCPGS No. 47-1049) [26]. Compared with the cubic phase NiO, the diffraction peaks are widened, and no obvious impurity peaks appear, indicating that the obtained nano NiO has a smaller particle size and higher purity. However, the diffraction peaks are still sharp, indicating good crystallization performance. The characteristic peaks of OD are located at 6.4°, 20.7°, 21.8°, 22.3°, and 24.6°. The peak positions of the NiO@CF/OD composite phase change thermal storage material are similar to that of OD, and no characteristic peak of NiO can be detected. We believe that the characteristic peak has been covered up and cannot be labeled because the pores of CF restrict the regular molecular arrangement of NiO during crystallization and the content of NiO is too small. No new peaks appeared in the NiO@CF/OD CPCMs, which means that OD and NiO@CF only participate in physical bonding, the crystal structure of OD has not changed, and the basic thermophysical properties of OD have been well preserved.

**Figure 4:**
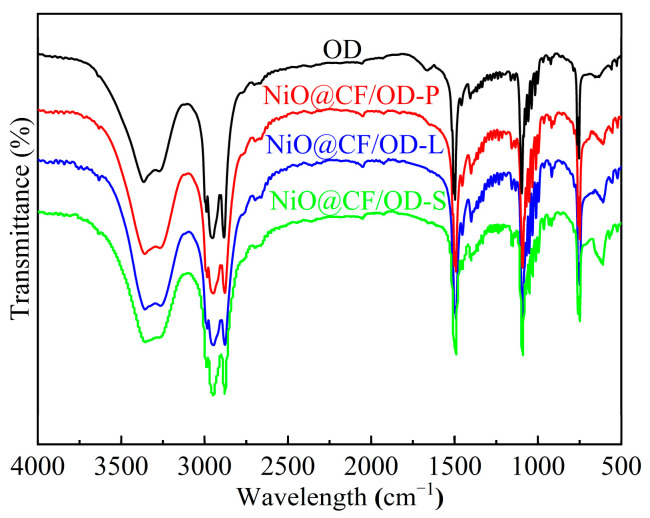
FTIR spectra of OD and NiO@CF/OD CPCMs.

The influence of CF and NiO on the chemical structure of OD was studied by FTIR spectroscopy. As seen in Figure 4, the peak at 1065 cm^−1^ corresponds to the stretching vibration of the C-O group [27,28]. The band at 609 cm^−1^ is due to the tensile vibration of Ni-O, which increases the strength of the Ni-O band. As shown in the spectra, the comparative observation of the spectra of NiO@CF/OD CPCMs and pure OD did not show any significant difference, and no new peaks appeared. It indicates an increase in NiO content in the composite material. Regarding the composite with CF and NiO nanosheets, the chemical structure of OD did not change and there was a high compatibility between OD and NiO@CF.

### 2.3. Mechanism Analysis of In Situ Generation of NiO Nanowalls

The growth mechanism of NiO has been a hot research topic in related fields. The formation mechanism of nano metal oxides is complex and largely depends on various factors such as time, temperature, hydrophobic and hydrophilic interactions, hydrogen bonding, static electricity, van der Waals forces, crystal plane attraction, dipole field, etc. The approximate growth process of NiO nanolayers in this article can be represented by Equations (1)–(3). Under hydrothermal conditions, urea hydrolysis releases NH_3_ and CO_2_ to form an alkaline solution. The weakly alkaline environment provides abundant nucleation sites and sufficient reaction time for the generation of precursor Ni(OH)_2_ nanosheets. The growth of Ni(OH)_2_ crystals is anisotropic, and based on the entropy reduction principle, Ni(OH)_2_ nanosheets are oriented to stack perpendicular to the (003) plane, thereby reducing their surface energy and electrostatic repulsion [29]. Ultimately, NiO nanowalls can be prepared by calcining Ni(OH)_2_ under air atmosphere.

(1) $${\mathrm{NH}}_{2}{\mathrm{CONH}}_{2}+H_{2}O\to {2\mathrm{NH}}_{3}+{\mathrm{CO}}_{2}$$

(2) $${\mathrm{Ni}}^{2+}+{2\mathrm{NH}}_{3}+{2H}_{2}O\to {\mathrm{Ni}(\mathrm{OH})}_{2}+{{2\mathrm{NH}}_{4}}^{+}$$

(3) $${\mathrm{Ni}(\mathrm{OH})}_{2}\to \mathrm{NiO}+H_{2}O$$

**Figure 5:**
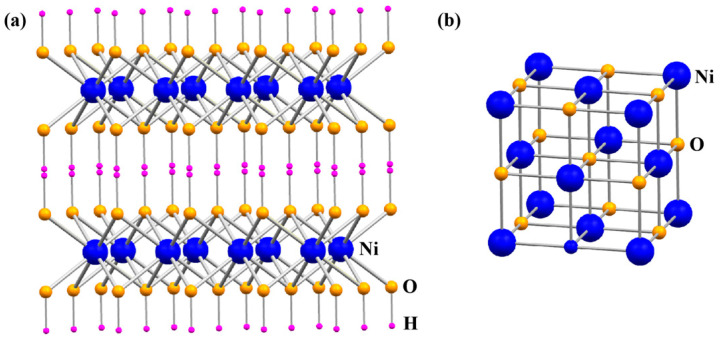
Crystal structure of (**a**) Ni(OH)_2_ and (**b**) cubic NiO.

In the process of in situ calcination to prepare nano NiO, the concentration of Ni(NO_3_)_2_·6H_2_O and urea directly affects the supersaturation of the precursor Ni(OH)_2_ in the solution. This is reflected in the crystal nucleation and growth direction and preferred orientation, ultimately determining the morphology and properties of nano NiO. During the reaction course, the microscopic morphology of NiO nanoparticles is mainly determined by the associated growth kinetics. In this paper, the obtained NiO nanowalls with various particle morphologies (for example, flaky and floral) can be theoretically analyzed from different nucleation processes and the growth of nanocrystalline crystals, triggered by different concentrations of reactants and the characteristics of the Ni(OH)_2_ crystal structure. Ni(OH)_2_ is generally considered to exist in two crystal forms, α-Ni(OH)_2_ and β-Ni(OH)_2_. Both crystal forms of Ni(OH)_2_ belong to the hexagonal crystal system, with the difference being that H_2_O molecules and other anions and cations are embedded in the interlayer of α-Ni(OH)_2_. α-Ni(OH)_2_ and β-Ni(OH)_2_ can be regarded as a layered stacking of NiO_2_, and the crystal structures of Ni(OH)_2_ and NiO are shown in Figure 5. In Ni(OH)_2_ crystals, Ni^2+^ ions on the (100) or (010) faces are connected by OH- ions, and a layer of Ni^2+^ on the z-axis adjacent to the (001) face is separated by upper and lower layers of OH-, which is a unique structure that gives Ni(OH)_2_ crystals the habit of growing in layers. In aqueous solutions, due to the presence of -OH, the concentration of negatively charged surface -OH on the (001) face is higher than that on the (010) and (100) faces, thus strengthening the interlayer repulsion, and thus Ni(OH)_2_ crystals tend to grow along the [010] and [100] directions; when the concentration of the reactants is lower, urea hydrolyzes to produce hydroxyl radical ions, which are the major contributing factor to the nanocrystalline growth. Hydroxyl radical ions provide a suitable environment for the nucleation and growth of Ni(OH)_2_ nanocrystals, and the supersaturation of Ni(OH)_2_ in the solution is small. Hence, the nucleation of the crystals is slow, and it is easy to form a thin film-like nanocurled product.

The different properties of solvents, such as viscosity, boiling point, polarity, etc., directly affect the nucleation and growth of particles. Solvents, inorganic additives, and surfactants can adsorb on a certain crystal plane and form different final forms by changing their surface energy. The surface energy of the initial crystalline nano products is relatively high. In order to reduce the surface energy of the products, the thin film-like structure is prone to agglomeration and curling to form particles. Each nanosheet is made up of an initial cluster of tiny nanoparticles. Hot spots in the solution due to hydrothermal properties, surfactants, and urea and organic compounds are the driving forces of initial nanoparticle nucleation, favoring the nucleation of precursor Ni(OH)_2_ nanoparticles and the subsequent growth of nanosheets and nanoflowers. As the concentration of reactants increases, the concentration of -OH in the solution increases, and excess -OH is inserted between the (001) planes, thereby expanding the (001) crystal plane spacing and increasing the z-axis size. The aggregated nanoparticles are oriented along the crystal faces and form flakes after assembly with uniform and slow precipitation conditions. When the concentration of the reactants reaches a certain value, the product will grow from a thin film structure to a lamellar form, and then the flakes undergo stochastic self-assembly and Osterwalder maturation processes to form a floral morphology. In addition, due to the increase in the concentrations of Ni(NO_3_)_2_-6H_2_O and urea, the supersaturation of the precursor Ni(OH)_2_ in the solution increases, and the size of the hexagonal flakes will decrease with the acceleration of the nucleation rate. α-Ni(OH)_2_ hexagonal flakes are slowly dehydrated during high-temperature calcination, thereby generating porous NiO. It can be seen that the microscopic morphology and structure of NiO nanoparticles can be regulated by simply adjusting the addition ratio of reactants. The mechanism in this paper can also be used to reveal the way that other nickel sources can used as raw materials for the preparation of Ni(OH)_2_ and NiO nanoparticles with different morphologies, and the method of preparing NiO nanoparticles in this paper can also be used to guide the synthesis of metal oxide nanoparticles with similar crystal structures.

### 2.4. Thermal Storage Properties

**Figure 6:**
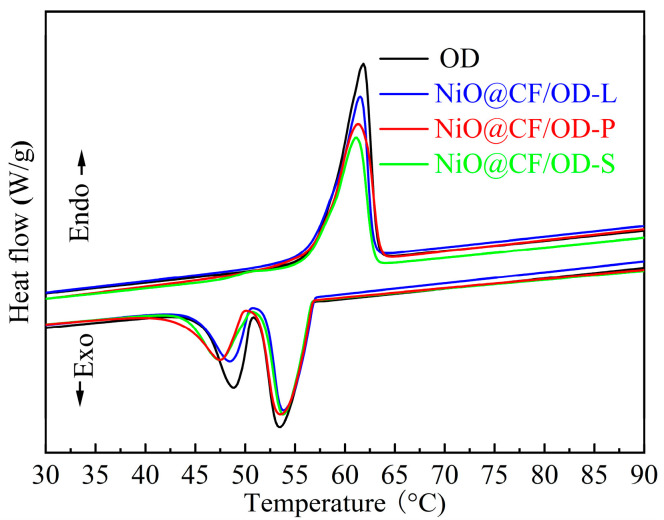
DSC curves of OD and NiO@CF/OD CPCMs.

Phase change temperature and enthalpies are the critical parameters for evaluating the thermal properties of phase change heat storage materials. Figure 6 shows the DSC melting and solidification curves of OD and NiO@CF/OD CPCMs. OD has only one endothermic peak in the melting process and two adjacent exothermic peaks in the solidification process. The endothermic and exothermic curves of NiO@CF/OD CPCMs are basically consistent with OD. Table 2 lists the phase change process data of the corresponding materials. The melting temperatures of NiO@CF/OD-P, NiO@CF/OD-L, and NiO@CF/OD-S CPCMs are 57.9 °C, 57.5 °C, and 56.9 °C, respectively, with relatively small changes in phase change temperature compared to OD. The enthalpies of melting and solidification of NiO@CF/OD-L CPCMs are 220.7 J/g and 218.3 J/g, which are 91.1% and 88.2% of OD, respectively. The latent heat of phase change of the NiO@CF/OD-S CPCMs is significantly reduced compared to the other two, with 180.2 J/g and 172.8 J/g, respectively, which are only 74.4% and 76.3% of OD. The main reason is that NiO in NiO@CF-S is too dense and has serious secondary growth, which leads to a great increase in the proportion of NiO@CF-S in the composite phase change thermal storage materials, thus significantly reducing the latent heat of phase change. The dense NiO nanosheets provide a larger contact area for the composite material while occupying a smaller mass ratio in the composite material, so their phase change enthalpy is slightly smaller than that of the CF/OD composite phase change heat storage materials.

### 2.5. Heat-Conducting Properties

**Figure 7:**
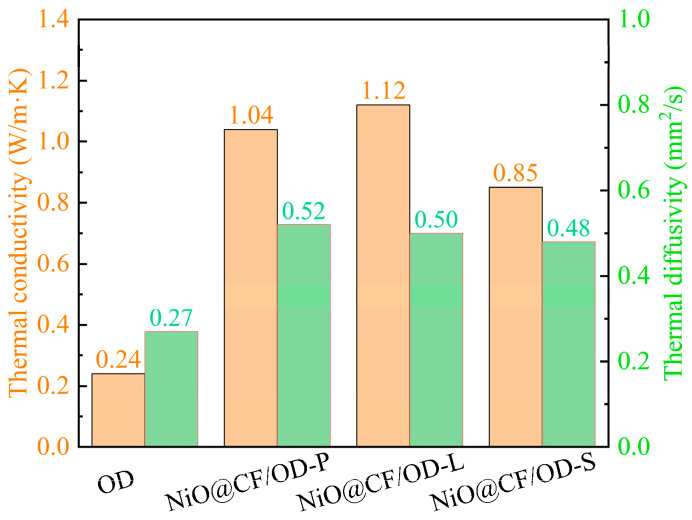
Thermal conductivity and thermal diffusivity of OD and NiO@CF/OD CPCMs.

**Figure 8:**
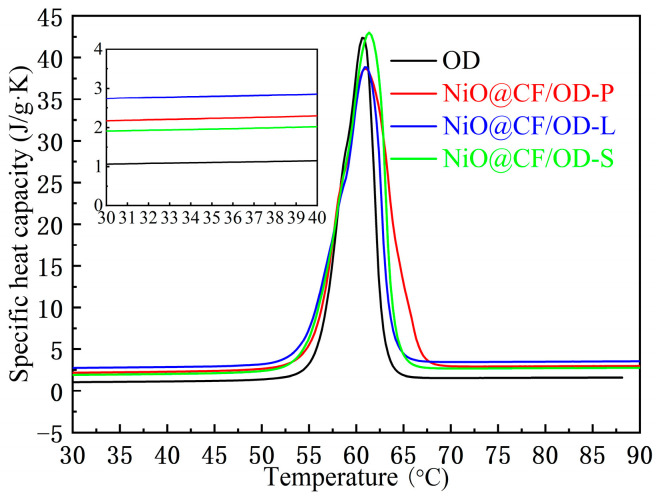
Specific heat capacity of OD and NiO@CF/OD CPCMs.

Figure 7 and Figure 8 demonstrate the thermal diffusivities, thermal conductivities, and specific heat capacities of OD and NiO@CF/OD CPCMs. The thermal conductivities of NiO@CF/OD-P, NiO@CF/OD-L, and NiO@CF/OD-S CPCMs are 1.04 W/m·K, 1.12 W/m·K, and 0.85 W/m·K, which are 333.3%, 366.7%, and 254.2% higher than OD, respectively. Specific heat capacity refers to the amount of heat absorbed or released by a unit mass object at a unit temperature without any phase or chemical changes. From Figure 8, the specific heat capacities of NiO@CF/OD-P NiO@CF/OD-L, and NiO@CF/OD-S CPCMs are 2.23 J/g·K, 2.80 J/g·K, and 1.96 J/g·K, respectively, which are significantly improved compared to the specific heat capacity of OD with 1.10 J/g·K. The large specific surface area and constructed pores of NiO nanosheets can adsorb a large amount of OD to increase the internal heat transfer area and improve heat transfer efficiency.

### 2.6. Thermal Reliability

**Figure 9:**
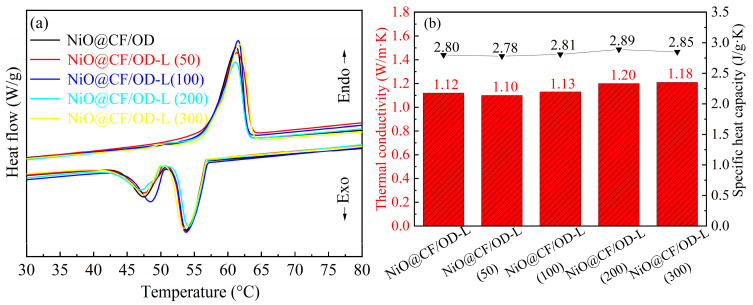
(**a**) DSC curves and (**b**) thermal conductivity and specific heat capacity of NiO@CF/OD CPCMs.

Excellent reversibility and durability are important properties of energy storage materials in practical applications, ensuring that their thermal storage performance does not undergo significant changes with prolonged use time. For this purpose, the NiO@CF/OD-L composite phase change thermal storage material was subjected to 300 cycles of 30–90–30 °C, and the samples were labeled as NiO@CF/OD-L (50), NiO@CF/OD-L (100), NiO@CF/OD-L (200), and NiO@CF/OD-L (300) in terms of the number of cycles. The phase change temperature, latent heat of phase change, thermal conductivity, and specific heat capacity of the CPCMs after the cycle times are shown in Figure 9. After 300 thermal cycles, the melting and solidification temperatures of the NiO@CF/OD-L composite phase change thermal storage material were 57.9 °C and 56.3 °C, respectively. In addition, NiO@CF/OD-L (300) still had a high energy storage density, with a latent heat of melting and latent heat of solidification of 215.6 and 179.2 J/g, which were 97.7% and 96.7% of the enthalpies of CPCMs, respectively. From Figure 9b, as the thermal cycle progresses, the thermal conductivity of NiO@CF/OD-L CPCMs first increases and then stabilizes at 1.21 W/m·K. During repeated heating and cooling processes, OD is fully filled into the mesopores of the NiO nanosheets. When the filling amount stabilizes, the thermal conductivity tends to stabilize.

### 2.7. Photothermal Conversion

**Figure 10:**
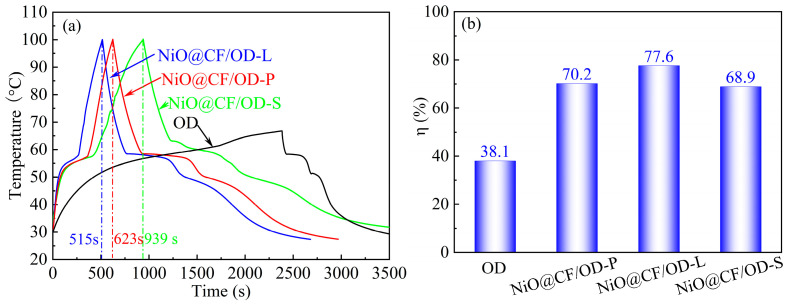
(**a**) Photothermal conversion curves and (**b**) photothermal conversion efficiency of OD and NiO@CF/OD CPCMs.

The thermal energy collection and storage properties of composite phase change thermal storage materials play an important role in light-heat utilization and energy management. In recent years, the use of functional composite materials with low phase transition temperatures for the direct collection and conversion of sunlight has received increasing attention. Figure 10a shows the temperature rise curves of OD and NiO@CF/OD CPCMs before and after irradiation by a sunlight simulator. The time required for the NiO@CF/OD-P, NiO@CF/OD-L, and NiO@CF/OD-S CPCMs to rise from 30 °C to 100 °C is 623 s, 515 s, and 939 s, respectively. NiO@CF/OD-L had the fastest temperature rise. OD cannot be heated to 100 °C due to its low thermal conductivity. In addition, the time required for the transformation of OD, NiO@CF/OD-P, NiO@CF/OD-L, and NiO@CF/OD-S composite phase change thermal storage materials was 1068 s, 83 s, 64 s, and 158 s respectively. Photothermal conversion is influenced by a combination of factors, such as the material microstructure, specific surface area, specific heat capacity, and thermal conductivity. The temperature rise rate of PCMs under light irradiation indirectly reflects the rate of heat transfer inside the PCMs. Combined with Figure 7, the temperature rise trend of NiO@CF/OD CPCMs aligns with its thermal conductivity characteristics. It can be seen that the addition of NiO@CF greatly improves the heat transfer and storage efficiency of OD. For OD, in addition to its low thermal conductivity, its white surface can greatly reflect irradiated light, which seriously limits the phase transition process. In contrast, the black surface of NiO@CF/OD CPCMs can trap photon energy and heat OD molecules, storing the heat energy inside OD through phase transition. Meanwhile, CF exhibits melting behavior at earlier and faster temperatures because it acts as a 3D skeleton for fast photon and heat transfer. The photothermal conversion efficiencies of the samples are shown in Figure 10b. The photothermal conversion efficiency (η) can be calculated using Equation (4).

η = m·ΔH/(P·S(t_0_ − t_e_)) (4)

where m is the mass, ΔH is the thermal enthalpy, P and S are the solar irradiation intensity (200 mW/cm^2^) and the surface area of the CPCMs, respectively, and t_0_ and t_e_ are the start and end times of the phase transition. The η of OD, NiO@CF/OD-P, NiO@CF/OD-L, and NiO@CF/OD-S CMCP are 38.1%, 70.2%, 77.6%, and 68.9%, respectively, which makes it very obvious that NiO@CF/OD can effectively capture radiated light and convert it into thermal energy. 

We compared the relevant literature on thermal properties of metal oxide-based CPCMs. Table 3 summarizes the data of the melting temperature (T_m_), thermal enthalpy, and thermal conductivity of each material. It is evident that various metal oxide materials have been incorporated into PCM matrices through diverse methodologies to enhance their thermal properties, resulting in markedly different effects. Cui [30] developed shape-stabilized MgO/PEG CPCMs, and bridged the gap between theory and practice in assessing the energetic impact of PCMs by using a model-driven approach to develop durable thermal energy storage materials with the desired phase transition properties. Cheng [31] prepared TiO_2_/PEG using the sol–gel method, with TiO_2_ acting as a supporting skeleton, maintaining the shape stability of the material and improving the thermal conductivity. In conclusion, the NiO@CF/OD CPCMs prepared in this study showed excellent performance in terms of structure and thermal properties, and therefore have a more reliable and long-lasting phase change capability in energy storage applications.

## 3. Experimental

### 3.1. Materials

1-Octadecanol (OD, C_18_H_38_O), urea (H_2_NCONH_2_), and nickel nitrate hexahydrate (Ni(NO_3_)_2_·6H_2_O) were purchased from Sinopharm Chemical Reagent Co., Ltd (Beijing, China). High-porosity MF was purchased from Chengdu Lotte Company (Chengdu, China), with a density of 8.9 mg/cm^3^.

### 3.2. Preparation of Carbon Foam

**Figure 11:**
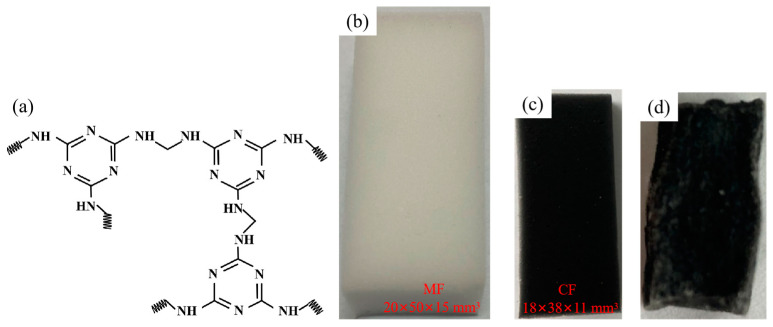
Structural formula of melamine foam (**a**), photographs of MF (**b**), photographs of CF (**c**), and NiO@CF/OD CPCMs (**d**).

Carbon foam (CF) was produced by high temperature carbonization of melamine foam (MF). Melamine foam is mainly composed of melamine, has a three-dimensional network structure, and contains rich nitrogen and oxygen elements. Its structural formula is shown in Figure 11a. Commercial MF contains some impurities, such as oil and grease, so it was necessary to clean it sufficiently before carbonizing the MF. The MF was divided into 2 × 5 × 1.5 cm^3^ and cleaned by thorough ultrasonication with deionized water and anhydrous ethanol for 1 h, respectively, followed by drying in a vacuum oven at 60 °C. The clean MF was then put into a high temperature oven, which used nitrogen as the protective gas. The carbonization temperature was set at 600 °C, the heating rate was 5 °C/min, and the carbonization time was 1 h. CF was obtained after the carbonization process. The preparation process and performance analysis of CF are detailed in another research paper by our research group [37].

### 3.3. Preparation of NiO@CF/OD CPCMs

**Figure 12:**
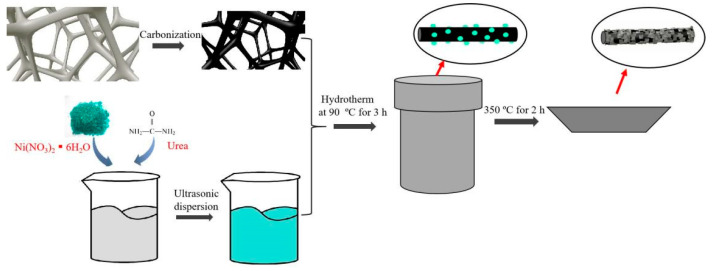
Fabrication procedures of NiO@CF.

Ni(NO_3_)_2_·6H_2_O (0.15 g) and urea (0.15 g) were dissolved completely in deionized water (20 mL) with ultrasonic treatment. CF with the size of 10 × 10 × 10 mm^3^ was completely submerged in the above solution and repeatedly squeezed so that it was repeatedly exposed to the solution. The CF was then put into a polytetrafluoroethylene reactor, and the reactor was placed under 90 °C hydrothermal conditions for 3 h. Under the synergistic catalysis of temperature and urea, the Ni(OH)_2_ precursor will be uniformly deposited on the surface of the foam carbon. The reactor was removed to cool naturally, and the CF was removed from the reactor and gently rinsed twice with alternating deionized water and ethanol. The samples were dried in an oven at 60 °C before use. The prepared samples were placed in a muffle furnace and heated to 350 °C at 10 °C/min for a 2 h annealing treatment to prepare NiO@CF materials. The NiO@CF preparation process is shown in Figure 12. NiO nanowalls with particles, lamellae, and spheres can be obtained by varying the mass ratio of Ni(NO_3_)_2_-6H_2_O and urea (1:2, 1:1, and 2:1). Samples were recorded as NiO@CF-P, NiO@CF-L, and NiO@CF-S.

NiO@CF was split into tiny pieces into a centrifuge tube with an appropriate amount of ethanol. NiO was separated out by high-speed centrifugation at 8000 r/min for 5 min, followed by collection of the material at the bottom of the centrifuge tube. Then, the above operation was repeated 3 times, and the final sample was dried in a drying oven at 110 °C to obtain pure nano NiO, which was recorded as NiO-P, NiO-L, and NiO-S, respectively.

NiO@CF/OD CPCMs were prepared by a melt blending and vacuum adsorption method. A sufficient amount of OD was weighed into a beaker and melted completely in a 90 °C water bath. The prepared NiO@CF sample was added to liquid OD to fully adsorb the molten OD. Since NiO@CF has a rich microporous structure, some of the internal pores could not be completely filled under atmospheric pressure, and it needs to be adsorbed twice under vacuum above the melting point. The beaker of NiO@CF/OD CPCMs was placed in a vacuum drying oven for vacuum adsorption at 90 °C for 6 h. The samples were dried and stored after cooling in the furnace and named NiO@CF/OD-P, NiO@CF/OD-L, and NiO@CF/OD-S, respectively.

### 3.4. Characterization

**Figure 13:**
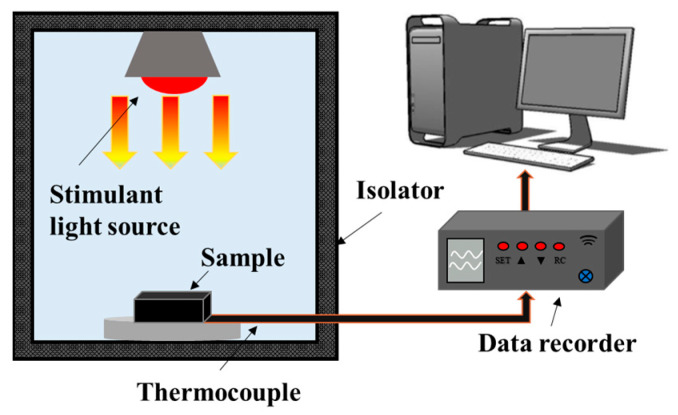
Schematic of photothermal energy conversion and storage test system.

Field emission scanning electron microscopy (SEM, JSM-7500F, JEOL, Tokyo, Japan) was used to observe the morphologies and analyze the material composition of the sample. An automatic surface area and pore analyzer (BET, ASAP 2460, Norcross, GA, USA) obtained the specific surface area and mesoporous structure data of samples. An X-ray diffractometer (XRD) can be used for phase identification and quantitative analysis of materials. A D8 ADVANCE X-ray diffractometer produced by Bruker, Bremen, Germany, was used to study the crystal structure of NiO. The diffraction source used was copper palladium (Cu Kα, λ = 1.5406 A), and the diffraction angle ranged from 5° < 2θ < 60°. Fourier transform infrared spectroscopy (FTIR), (Nicolet6700, Madison, WI, USA), was used to analyze the carbonization process of melamine foam and to test the molecular structure of composite phase change thermal storage materials. The test wavelength range was 5000–350 cm^−1^, with a resolution of 4 cm^−1^. The phase change temperature and enthalpies of the samples were determined by differential scanning calorimetry (DSC, NETZSCH, Selb, Germany) with the temperature range set to 30–80 °C and a heating rate of 5 °C/min. A laser thermal conductivity meter (LFA457, Netzsch, Selb, Germany) was used to measure the thermal diffusivity and thermal conductivity of the samples. As shown in Figure 13, the photothermal conversion test system consists of a thermal insulation unit, hernia lamp (CEL-NP2000, Ceaulioht, Beijing, China), T-type thermocouples, and a data acquisition system.

## 4. Conclusions

This study investigates the formation of NiO nanowalls with distinct array morphologies on the surface of CF fibers through in situ calcination, and NiO@CF/OD CPCMs were prepared by melt blending and vacuum adsorption. The well-arranged NiO nanowalls ensure the close combination of CF and OD, effectively solving the problem of leakage in the liquid state of CPCMs, and is conducive to the overall enhancement of the thermal performance of the NiO@CF/OD CPCMs. The melting enthalpy and solidification enthalpy of NiO@CF/OD-L CPCMs were 220.7 J/g and 218.3 J/g, which are 91.1% and 88.2% of OD, respectively, NiO@CF/OD CPCMs have a suitable phase change temperature and high phase change enthalpy. After 300 thermal cycles, the melting and solidification temperatures of NiO@CF/OD-L CPCMs were 57.9 °C and 56.3 °C, respectively, and the latent heat of melting and solidification were 215.6 J/g and 179.2 J/g, which were 97.7% and 96.7% of the enthalpy of the composite material before the thermal cycle. Moreover, NiO@CF/OD CPCMs have excellent thermal reliability and thermal cycling stability, and exhibit an excellent photothermal conversion efficiency, at 77.6%. This work provides a novel approach towards developing composite phase change thermal storage materials with superior thermal performance, which can be used for low-temperature thermal energy conversion and storage.

## Figures and Tables

**Table 1:** Porosity characteristics of NiO@CF.

Samples	Specific Surface Area m^2^/g	Pore Volume cm^3^/g	Average Pore Size nm
NiO@CF-P	176.2	0.09	5.69
NiO@CF-L	101.9	0.07	2.86
NiO@CF-S	80.5	0.05	2.41

**Table 2:** Phase change temperatures and thermal enthalpies of OD and NiO@CF/OD CPCMs.

Samples	Melting		Solidifying	
	T_m_ (°C)	ΔH_m_ (J/g)	T_s_ (°C)	ΔH_s_ (J/g)
OD	57.7	242.2	56.7	210.1
NiO@CF/OD-P	57.9	218.9	56.5	189.0
NiO@CF/OD-L	57.5	220.7	57.6	185.3
NiO@CF/OD-S	56.9	180.2	57.1	160.3

**Table 3:** Comparison of thermal properties of CPCMs in related references.

Filler	Methods	Matrix	T_m_ °C	Thermal Enthalpy (J/g)	Thermal Conductivity (W/m K)	Ref.
Mesoporous MgO	Evaporative pyrolysis	PEG1000	31.5	100.7	--	[30]
TiO_2_	Sol–gel	PEG	55	123.3	0.39	[31]
CuS/ZnO	Calcination	PA	75.2	155	0.45	[32]
CuO	Direct addition	Lauryl alcohol-Capric acid	8.7	159.1	0.17	[33]
SiO_2_/TiO_2_	Sol–gel	Paraffin	29.0	93.7	0.2	[34]
Hollow porous Co_3_O_4_-EG	In-suit	Steric acid	69.4	192.8	1.26	[35]
Nano TiO_2_/carbon nanofiber	Direct addition	OD	57.6	209.3	0.43	[36]
NiO@CF	Calcination	OD	56.5	208.3	1.12	This work

## Data Availability

The original contributions presented in the study are included in the article; further inquiries can be directed to the corresponding author.
